# Growth kinetics of *Chlamydia trachomatis* in primary human Sertoli cells

**DOI:** 10.1038/s41598-019-42396-3

**Published:** 2019-04-10

**Authors:** Simone Filardo, Rachel J. Skilton, Colette E. O’Neill, Marisa Di Pietro, Rosa Sessa, Ian N. Clarke

**Affiliations:** 1 Department of Public Health and Infectious Diseases, Section of Microbiology, Sapienza University, Rome, Italy; 2 Molecular Microbiology Group, Faculty of Medicine, University of Southampton, Southampton General Hospital, Southampton, UK

## Abstract

*Chlamydia trachomatis* (Ct) is the leading cause of bacterial sexually transmitted infections worldwide and has been associated with male infertility. Recently, it was hypothesized that Ct may infect the epithelium of the seminiferous tubule, formed by Sertoli cells, thus leading to impaired spermatogenesis. To date, there is a lack of data on Ct infection of the seminiferous epithelium; therefore, we aimed to characterize, for the first time, an *in vitro* infection model of primary human Sertoli cells. We compared Ct inclusion size, morphology and growth kinetics with those in McCoy cells and we studied F-actin fibres, Vimentin-based intermediate filaments and α-tubulin microtubules in Sertoli and McCoy cells. Our main finding highlighted the ability of Ct to infect Sertoli cells, although with a unique growth profile and the inability to exit host cells. Furthermore, we observed alterations in the cytoskeletal fibres of infected Sertoli cells. Our results suggest that Ct struggles to generate a productive infection in Sertoli cells, limiting its dissemination in the host. Nevertheless, the adverse effect on the cytoskeleton supports the notion that Ct may compromise the blood-testis barrier, impairing spermatogenesis.

## Introduction

*C. trachomatis* is an obligate intracellular pathogen characterized by a distinctive developmental cycle, alternating between two morphologically and functionally distinct forms, the elementary body (EB), the extracellular infectious form, and the replicative body (RB), the intracellular replicative form^1,2^.

*C. trachomatis* is the leading cause of bacterial sexually transmitted infections worldwide, with more than 130 million new cases per year^3^. In men, *C. trachomatis* is a major cause of urethritis, with up to 42% of all cases of non-gonococcal urethritis attributed to a chlamydial infection^4^. However, its genuine prevalence remains unknown and probably underestimated, since 50% of chlamydial infections in men are asymptomatic and, hence, undetected and untreated, leading to potential complications^3^. Indeed, it is now widely accepted that ascending chlamydial infections are involved in the etiopathogenesis of acute or chronic epididymitis^5,6^ and chlamydial antigens have also been detected in men with epididymo-orchitis, suggesting a causative link between *C. trachomatis* and infection of the testis^5,6^ that may contribute to the development of infertility.

The etiopathogenesis of infertility in men is characterised by different causes, ranging from genetic disorders to testicular dysfunction, obstructive azoospermia, varicocele and hormonal imbalance^7,8^. In more than half of all cases, the aetiology is unknown, and amongst all the potential risk factors, *C. trachomatis* has been the target of several studies that suggested an association between this pathogen and male infertility^8–15^. To explain its involvement, several potential pathophysiological mechanisms have been investigated and, amongst them, one theory postulates that the infection of the seminiferous tubule epithelium by *C. trachomatis* might lead to inflammatory damage and, hence, result in impaired spermatogenesis^8,9^. Interestingly, a report analysing a model of *C. muridarum* infection in male C57BL/6 mice demonstrated that this pathogen severely affects the seminiferous tubules, formed by Sertoli cells, leading to compromised spermatogenesis with reduced sperm count, motility and altered morphology of mature spermatozoa^16^.

Sertoli cells are key elements for the development of germ cells, since they finely regulate the spermatogenetic process through either the secretion of endocrine and paracrine mediators or by guiding germ cells from the basal to the adluminal compartment of the seminiferous tubule, that is physically divided by the blood-testis barrier (BTB)^17,18^. To accommodate the entry of spermatocytes into the adluminal compartment, existing junctions above them must transiently disassemble, leading to changes in protein-protein interactions^17^. The restructuring of the BTB is a complex process that is mainly supported by the Sertoli cell cytoskeleton, and, as such, the maintenance of its structural integrity is essential for the generation of mature functional spermatozoa^17,19,20^.

The idea that *C. trachomatis* may indirectly contribute to the etiopathogenesis of male infertility by infecting Sertoli cells is intriguing and, to date, there is a lack of data on these interactions.

Therefore, the aim of this study was to characterize *C. trachomatis* infection of Sertoli cells and, to do so, we developed an *in vitro* infection model of primary human Sertoli cells to mimic the physiology of the human testis. Also, we analysed the changes in the integrity of Sertoli cell cytoskeleton network following *C. trachomatis* infection, that may lead to a loss of functionality or disruption of the BTB.

## Results

### *In vitro* model of primary human Sertoli cells

We first analysed the growth behaviour of the human cell line selected for analysis. The primary human Sertoli cells were frozen at the 3^rd^ passage after isolation and were cultured up to the 9^th^ passage on 80 cm^2^ or 175 cm^2^ cell culture flasks. Cells were passaged every 7 days at a confluence level of 70–80%. The average yield on 80 cm^2^ flasks was 7.25 × 10^5^ ± 3.9 × 10^4^ cells/flask up to the 6^th^ passage, where upon it decreased by half by the 9^th^ passage (data not shown). Therefore, all our experiments were carried on primary human Sertoli cells at the 5^th^ passage after isolation. Primary human Sertoli cells grown on coverslips possess a thin and wide cytoplasm with irregular morphology and are approximately ten times the size of McCoy cells.

### The morphological phenotype of *C. trachomatis* inclusions in primary human Sertoli cells

**Figure 1:**
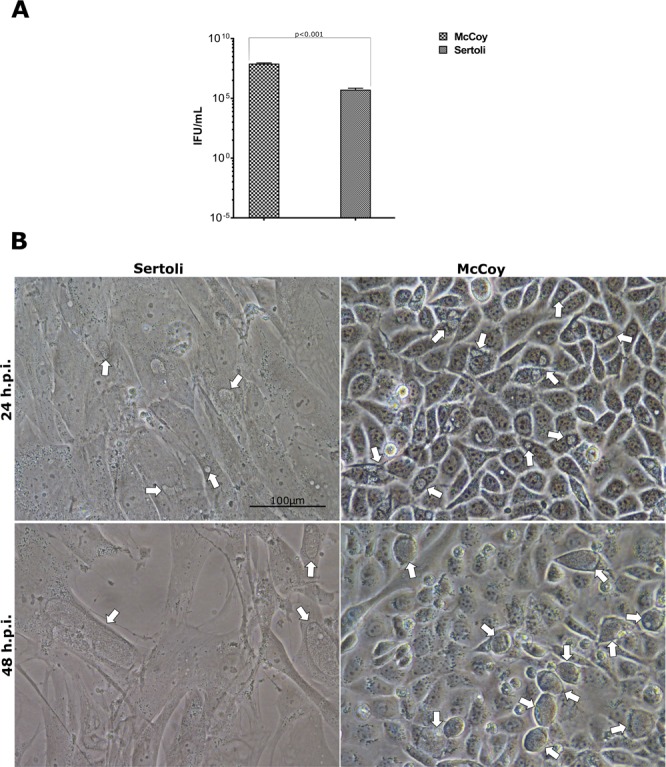
Comparison of *C. trachomatis* infectivity and morphological phenotype in primary human Sertoli and McCoy cells. (**A**) Yield of *C. trachomatis* D/UW-3/CX infection of primary human Sertoli and McCoy cells at a MOI = 1.0, expressed as means ± SD of four replicates from two independent experiments; (**B**) Phase-contrast micrographs of *C. trachomatis* inclusions in primary human Sertoli and McCoy cells at 24 and 48 hours post infection. Representative images of ten microscope fields are shown. Arrows point to chlamydial inclusions.

The infection of both Sertoli and McCoy cell monolayers with the same Multiplicity of Infection (MOI = 1.0) of *C. trachomatis* (originally titrated in McCoy cells) resulted in reduction of the number of inclusion forming units (IFUs) by approximately 150 times lower in Sertoli cells as compared to McCoy cells (5.3 × 10^5^ ± 1.49 × 10^5^ IFU/mL and 8.03 × 10^7^ ± 1.01 × 10^7^ IFU/mL respectively, *p* < 0.001), with a Sertoli/McCoy ratio of 0.0069 ± 0.0028 (Fig. 1A).

Furthermore, at 24 hours post infection (hpi) and, in particular, at 48 hpi, *C. trachomatis* inclusions were bigger in primary human Sertoli cells than in McCoy cells (335.1 ± 215µm^2^ and 143.8 ± 37.43 µm^2^ at 24 hpi.; 2334 ± 1319.5 µm^2^ and 742.2 ± 214.73 µm^2^ at 48 hpi, *p* = 0.22 and *p* = 0.23 respectively) showing irregular morphology, as demonstrated in Fig. 1B. Nevertheless, differences in the size of chlamydial inclusions were not statistically significant.

### Growth kinetics of *C. trachomatis* in primary human Sertoli cells

To characterize the growth phenotype of *C. trachomatis* in primary human Sertoli cells, we sampled multiple, regular time-points across the duration of chlamydial developmental cycle for infectivity, DNA replication and inclusion size.

**Figure 2:**
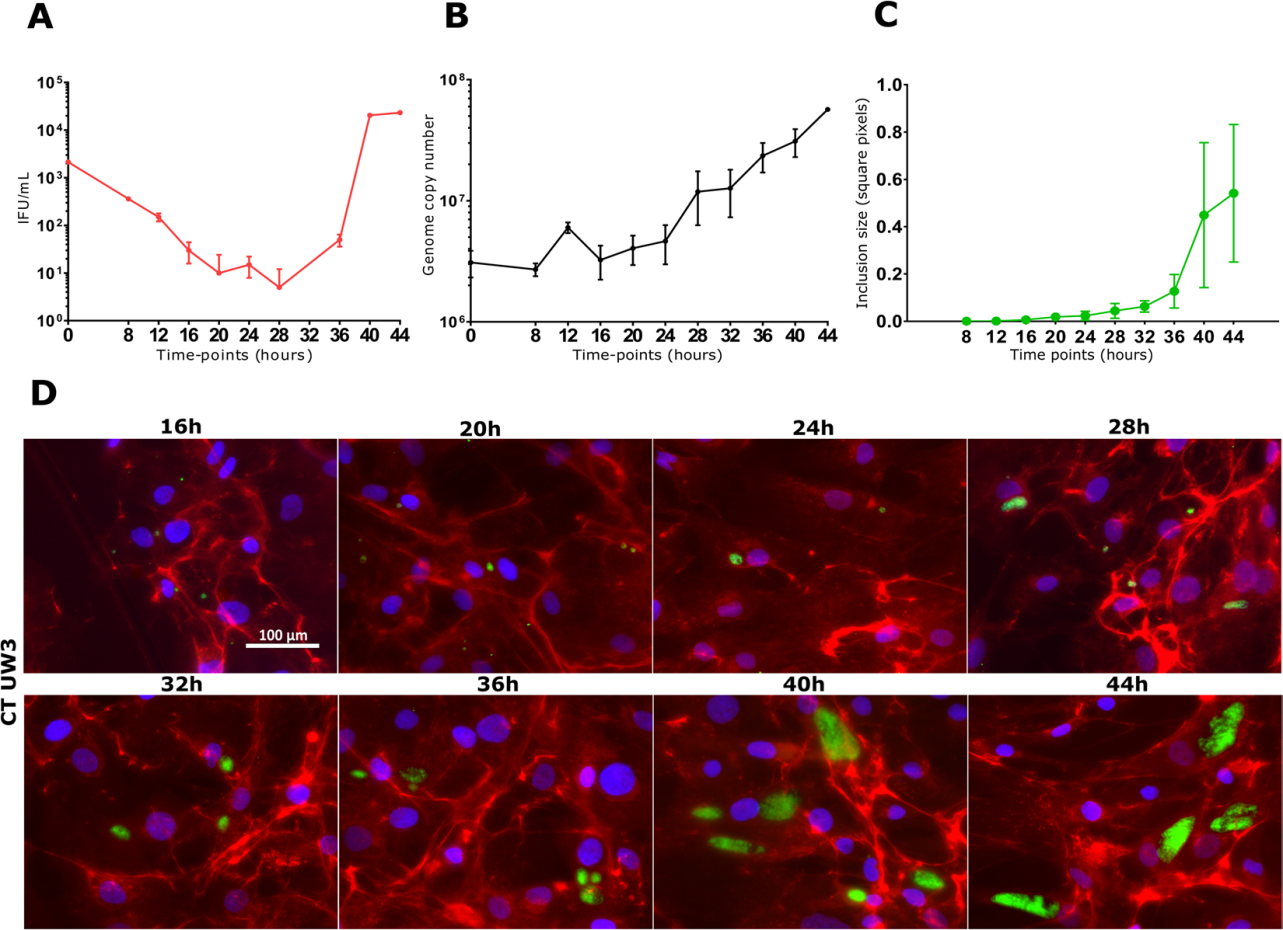
Growth kinetics of *C. trachomatis* in primary human Sertoli cells. Infectivity profile (**A**), chromosomal replication (**B**), quantification of the inclusion size (**C**) and fluorescence micrographs (**D**). Infected primary human Sertoli cells were removed for analysis at 4 hourly intervals and then titrated to assess the quantity of IFUs and the relative number of chlamydial genomes by qPCR. Values are expressed as means ± SD of four replicates from two independent experiments. Chlamydial inclusions were visualized by indirect immunofluorescence with species-specific anti-MOMP monoclonal antibodies (Mab6ciii). Representative images of ten chlamydial inclusion per time-point are shown. Mean and standard deviation of inclusion size, expressed as square pixels, were measured from fluorescence micrographs using ImageJ software.

The one-step infectivity growth curve showed a relatively long eclipse period for *C. trachomatis* in human Sertoli cells, with infectious EBs appearing exclusively after 36 hours post infection (Fig. 2A). By contrast, chromosomal DNA replication, measured by qPCR, showed a constant and gradual increase in genome copy number over the course of the experiment (Fig. 2B), not correlating with the sharp increase in infectivity after 36 hpi. The generation time for *C. trachomatis* in primary human Sertoli cells was 5 hours and 34 minutes.

The ‘inclusion size’ growth curve showed chlamydial inclusions growing gradually up to 32 hpi, whereupon it showed a sudden increase in size after 36 hpi, as evidenced by Fig. 2C,D.

### *C. trachomatis* lytic exit from primary human Sertoli cells

To investigate the exit of *C. trachomatis* from primary human Sertoli cells, we observed infected cells every 24 up to 96 hpi.

**Figure 3:**
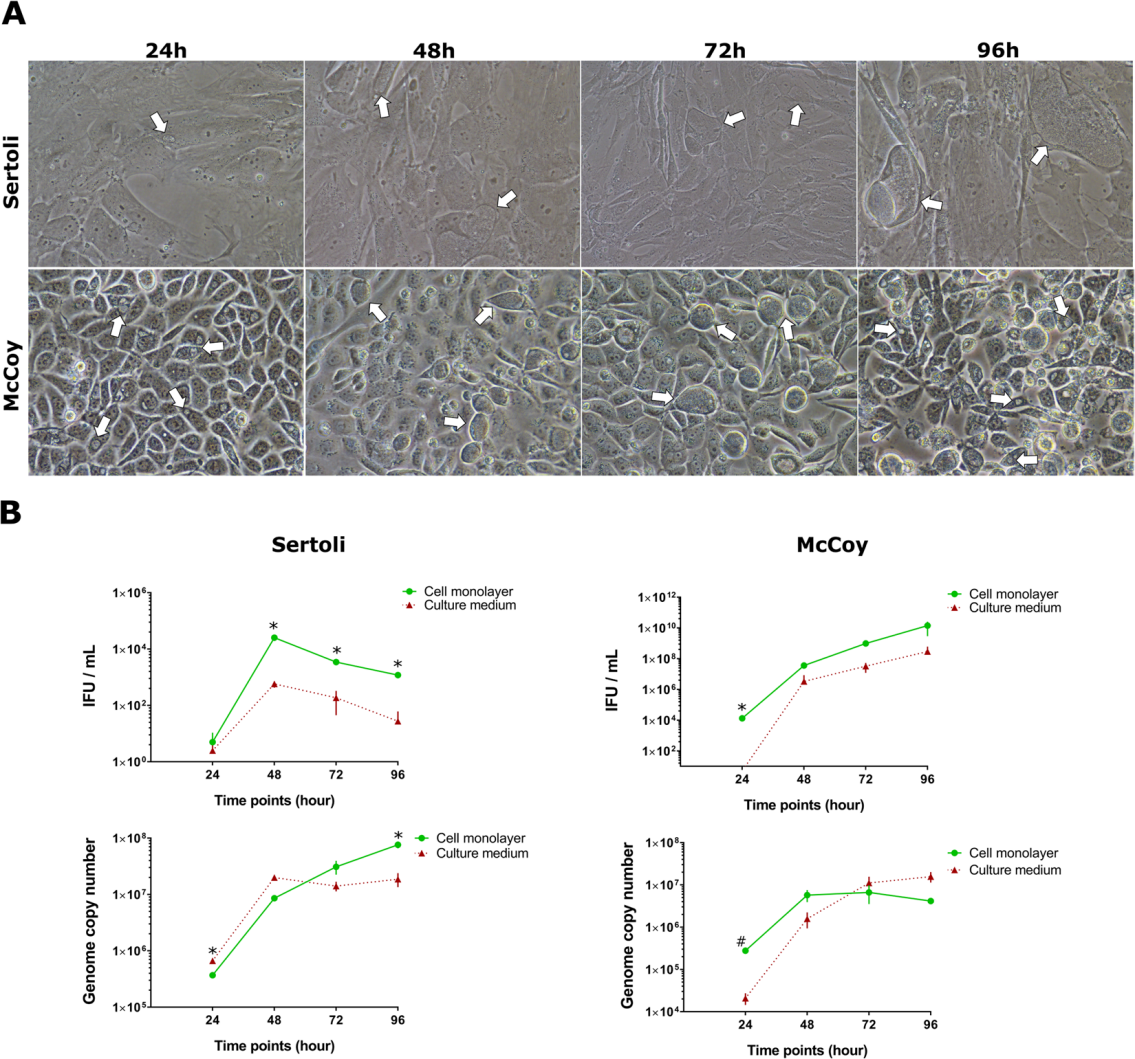
Reduced cell lysis of *C. trachomatis* infected primary human Sertoli compared with McCoy cells. Phase contrast micrographs (**A**) and one-step infectivity growth profiles and chromosome replication (**B**) of *C. trachomatis* in Sertoli and McCoy cells at 24 hourly intervals up to 96 hours post infection. Values are expressed as means ± SD of four replicates from two independent experiments. Representative images of ten chlamydial inclusions per condition are shown. White arrows indicate inclusions. *cell monolayer vs culture medium *p* < 0.01; ^#^cell monolayer vs culture medium *p* < 0.05.

Remarkably, *C. trachomatis* did not exhibit the ability to exit from host cells in primary human Sertoli cells, revealing the lack of cell lysis in Sertoli cells at 96 hpi (Fig. 3A). In fact, the one-step infectivity growth curve showed that the majority of infectious EBs were retained in the Sertoli cell monolayer (Fig. 3B). Furthermore, the number of infectious EBs decreased after 48 hpi in both Sertoli cell monolayer and culture medium (*p* < 0.01). By contrast, the qPCR assay demonstrated continuous chromosomal DNA replication up to 96 hours post infection.

In McCoy cells, *C. trachomatis* infectivity constantly increased from 48 to 96 hpi and infectious EBs were released into the culture medium, as shown in Fig. 3B. As expected, the qPCR assay showed the same trend as seen in primary human Sertoli cells.

### Confocal analysis of Sertoli cell cytoskeleton following *C. trachomatis* infection

**Figure 4:**
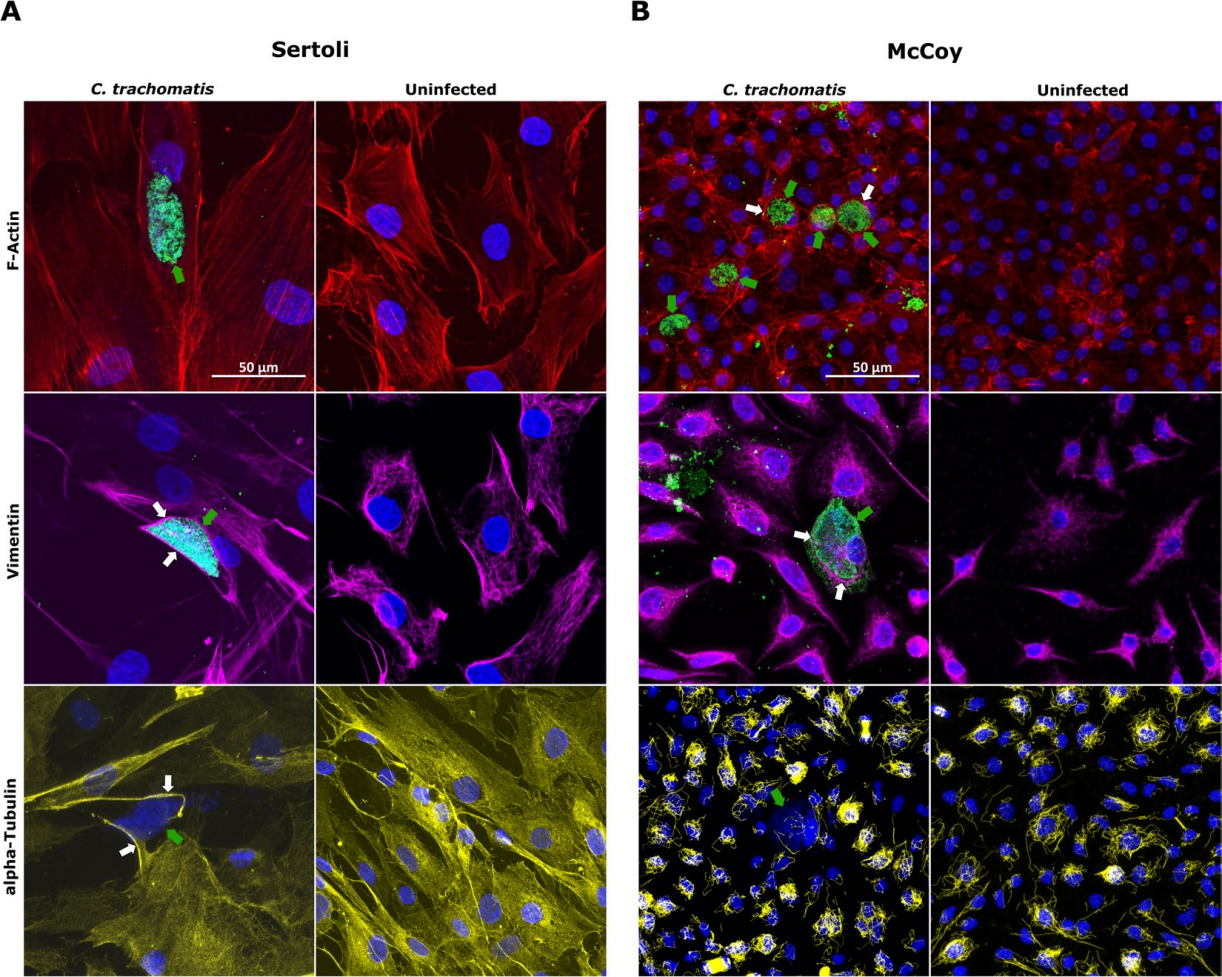
Confocal analysis of cell cytoskeleton in primary human Sertoli and McCoy cells infected by *C. trachomatis*. Laser scanning confocal micrographs of F-actin microfilaments, Vimentin-based Intermediate Filaments and α-tubulin microtubules in primary human Sertoli (**A**) and McCoy (**B**) cells infected with *C. trachomatis*. Representative images of ten chlamydial inclusions are shown (100X magnification). White arrows point to assemblies of cytoskeleton fibres lining chlamydial inclusions. Green arrows point to *C. trachomatis* inclusions.

In Sertoli cells, we found that the morphology of stress fibres and cortical actin in infected cells was not overly affected by *C. trachomatis*, whereas we observed the re-organization of Vimentin-based IFs and microtubules in thick fibres surrounding the inclusion (Fig. 4A).

By contrast, in McCoy cells we observed the recruitment of F-actin fibres to form a ring-shaped structure around the inclusion, as well as a similar restructuring of Vimentin-based IFs (Fig. 4B). However, the microtubule network was not perturbed by the presence of *C. trachomatis* inclusions.

## Discussion

In this study we characterized a novel *in vitro* infection model of *C. trachomatis* in primary human Sertoli cells. Our main finding demonstrated the ability of *C. trachomatis* to infect and replicate in primary human Sertoli cells, although with a much lower efficiency than in McCoy cells, which are considered to be the gold standard of Chlamydia laboratory culture because of their permissiveness to all strains of *C. trachomatis*^21^.

More importantly, the growth kinetics of *C. trachomatis* in primary human Sertoli cells growing under optimal conditions revealed a distinct profile, namely the very long eclipse period and the late appearances of infectious EBs toward the end of the developmental cycle. This was accompanied by a steady increase in genomic DNA copy number during the entire duration of the *C. trachomatis* developmental cycle, as evidenced by the qPCR analysis. By contrast, in McCoy cells the exponential phase of the infectivity growth curve is known to happen much earlier, at 20–22 hours post infection^22,23^. A possible explanation of this phenomenon may lie in a delayed differentiation of chlamydial RBs, that keep replicating as suggested by the qPCR, possibly the consequence of a hostile cellular environment that may partially arrest or contain chlamydial intracellular growth.

Further evidence that *C. trachomatis* struggles to infect and develop inside human Sertoli cells is also provided by the absence of cell lysis at the end of the developmental cycle, as evidenced by the retention of the majority of EBs within host cells, and by the constant decrease in the number of infectious EBs after 48 hours up to 96 hours post infection. This is intriguing and may suggest that *C. trachomatis* infection in Sertoli cells might lead to a persistent or chronic state.

All the above evidence suggests that *C. trachomatis*, despite being able to generate a productive infection of human Sertoli cells, is limited in its further dissemination within the human testis and potentially leads to long-term damage of Sertoli cell function and structure.

In fact, by analysing the subcellular localization and integrity of the main components of cell cytoskeleton, namely F-Actin fibres, Vimentin-based intermediate filaments and α-tubulin microtubules, we observed that the development of a *C. trachomatis* inclusion into a human Sertoli cell led to alteration in its cytoskeleton. Recent studies have shown that actin and microtubule-based cytoskeleton in Sertoli cells plays a crucial role to preserve the homeostasis of the BTB^20^. Our observation that Vimentin-based intermediate filaments and α-tubulin microtubules were re-organized in thick fibres surrounding the chlamydial inclusion, hints at the intriguing possibility that *C. trachomatis* might adversely affect the integrity of the BTB, and, hence, impair the spermatogenesis. Nevertheless, to confirm our preliminary observations, a polarized three-dimensional cell culture model of human Sertoli cells infected with *C. trachomatis* is needed for a more in-depth investigation of the different junction types forming the BTB.

The main strength of our study lies in the design of an *in vitro* infection model that utilizes a primary human Sertoli cell line, for better mimicking the physiology of their *in vivo* counterpart, infected with *C. trachomatis* serovar D, one of the most prevalent strains in men with urethritis^24–26^. In addition, to address potential variations between different cell preparations, we utilized a well characterized and commercially available primary human Sertoli cell line with optimised growth media and culture conditions. To minimize the variability of results, all our experiments were performed at the 5^th^ passage after isolation, following the observation that primary human Sertoli cells underwent significant morphological changes after the 7^th^ or 8^th^ passage.

Our future goal will be to set up a *C. trachomatis* infection model using a permanently established human Sertoli cell line to be compared to primary cells; this cell line will be widely shared to encourage research in this field. Furthermore, it will be helpful to investigate the morphology and development of *C. trachomatis* inclusions in Sertoli cells through more advanced imaging techniques such as scanning electron microscopy, to clarify whether the slow developmental cycle and the inability to exit host cells might be a mechanism of persistence.

## Methods

### Cell cultures and culture conditions

McCoy cell line (ECACC, Public Health England, UK, catalogue number 90010305) was cultured in Dulbecco’s Modified Eagle Medium (DMEM, Gibco™, USA) supplemented with 10% (v/v) foetal calf serum (FCS) (complete medium), at 37 °C in humidified atmosphere with 5% CO_2_.

The primary human Sertoli cell line that we utilized was purchased from Lonza, USA (product code MM-HSE-2305, lot number 360806141) and was harvested on 14th June 2011. Sertoli cells were cultured in Dulbecco’s Modified Eagle Medium/Ham’s Nutrient Mixture F12 (1:1), with L-glutamine and HEPES (DMEM/F12, Gibco™, USA), supplemented with 10% (v/v) FCS at 37 °C in humidified atmosphere with 5% CO_2_.

### Propagation and titration of *C. trachomatis*

*C. trachomatis* serovar D strain UW3 ATCC VR-855 was propagated in McCoy cells as previously described^27^. Briefly, confluent McCoy cell monolayers grown in 25 cm2 flasks were infected with chlamydial EBs by centrifugation at 754xg for 30 min and harvested by scraping after 36 hours of incubation. The suspension containing Chlamydial EBs was, then, added to equal volumes of 4X Sucrose Phosphate (4SP) buffer and stored at −80 °C.

For measuring *C. trachomatis* infectivity, confluent McCoy cell monolayers were infected with 10-fold dilutions of *C. trachomatis* EB suspension and, 36-hours post-infection, were fixed in 96% ice cold methanol. Chlamydial inclusions were blue-stained by using a mouse monoclonal antibody against genus-specific chlamydial LPS (Mab29, The Chlamydia Biobank, UK) followed by an anti-mouse antibody conjugated with β-galactosidase (Millipore, USA) and incubated with a X-Gal staining solution as previously described^27^. Blu-stained inclusions were visualized and counted by using a bright-field inverted microscope (200X magnification).

### Efficiency of *C. trachomatis* infection in Sertoli and McCoy cells

The efficiency of *C. trachomatis* infection in primary human Sertoli and McCoy cells was investigated by infecting confluent cell monolayers grown in 96-well trays with *C. trachomatis* at a MOI = 1.0 and by counting the number of IFUs/mL in both cell lines as above described.

### One-step growth curve of *C. trachomatis* in primary Sertoli cells

The one-step growth curve of *C. trachomatis* in primary human Sertoli cells was performed as previously described^27^. Briefly, confluent primary Sertoli cell monolayers were infected with *C. trachomatis* at a MOI = 1.0 in 24 and in 96 well trays for the infectivity assay and the qPCR analysis, respectively. Every 4 hours, from 8 to 44 hpi, infected cells were harvested and stored at −80 °C. At each time point, a 96-well tray was also stored at −80 °C for nucleic acid extraction.

### *C. trachomatis* inclusion lysis time-course in primary Sertoli cells

To observe the lysis of *C. trachomatis* inclusions and the release of infectious EBs in primary Sertoli cells, confluent cell monolayers were infected with *C. trachomatis* at a MOI = 1.0 in 24 and in 96 well trays as for the one-step growth curve as above described. The infection was stopped at 24, 48, 72 and 96 hpi and, at each time point, cell monolayers and supernatants were collected and stored at −80 °C.

### Infectivity assay

To assess infectivity at each time-point for the one-step growth curve and the inclusion lysis time-course in primary human Sertoli cells, *C. trachomatis* EB suspensions were titrated as above described.

### Real-time quantitative PCR analysis

Chromosomal DNA quantification at each time point of the one-step growth curve and the inclusion lysis time-course was determined as previously described^28^. The primers CM_omcB_F (5′-GGAGATCCTATGAACAAACTCATC-3′), CM_omcB_R (5′-TTTCGCTTTGGTGTCAGCTA-3′) and the probe CM_omcB_Probe (5′-FAM-CGCCACACTAGTCACCGCGAA-TAMRA-3′) were used for amplifying a conserved region in the omcB gene. The qPCR was performed in a VIIA7 Real-Time PCR system (Applied Biosystem).

### Confocal microscopy

Primary Sertoli cells grown at 60% confluence on glass coverslips in 24 well trays were infected with *C. trachomatis* at a MOI = 1.0 in the absence of cycloheximide, fixed in 4% paraformaldehyde and permeabilised using saponin buffer. Then, cell monolayers were stained for confocal microscopy as previously described^27^. The antibodies used were: *i*. a primary mouse monoclonal antibody against species-specific MOMP (Mab6ciii, The Chlamydia Biobank, UK, Cat. No. #CT602) (1:1000 dilution) combined with an anti-mouse-Alexa Fluor™ 488 conjugate secondary antibody (Invitrogen™, USA, Cat. No. A11001) (1:2000 dilution); *ii. a*n anti-Vimentin mouse monoclonal antibody-eFluor™ 660 conjugate (Affymetrix eBioscience, USA, Cat. No. 50-9897-82) (2.5 µg/mL); *iii*. a primary anti-α tubulin mouse monoclonal antibody (Invitrogen™, USA, Cat. No. 62204) (1:100 dilution) combined with an anti-mouse-Alexa Fluor Plus 647 conjugate secondary antibody (Invitrogen™, USA, Cat. No. A32728) (1:1000 dilution). Alexa Fluor™-594 Phalloidin (Invitrogen™, USA, Cat. No. A12381) was used for high-affinity labelling of F-Actin microfilaments. Nuclei were counterstained with 1 µg/mL DAPI (Fisher Scientific, USA). Images were captured using a Leica TCP SP8 confocal microscope at 1000X magnification. Confocal image processing and inclusion size measurement from fluorescence microscope images (µm2) were executed in ImageJ software (NIH, USA, version 1.8.0_112).

### Statistical analysis

All values are expressed as means ± standard deviation (SD) of two to four replicates from at least two independent experiments. Comparisons of means were performed by using a two-tailed Student *t-*test for independent samples. The single or multiple inference significance level was set to 5%. The *C. trachomatis* infection efficiency in Sertoli and McCoy cells, the one step growth curves, the chlamydial inclusion lysis time-course and the chromosomal replication graphs, as well as all statistical calculations, were produced in GraphPad Prism software (GraphPad Software, USA, version 7.0.3.0).

## Data Availability

The datasets generated and/or analysed during the current study are available from the corresponding author on request.

## References

[1] Sessa, R., *et al*. Lactobacilli-lactoferrin interplay in *Chlamydia trachomatis* infection. *Pathog Dis*. **75**(5), 10.1093/femspd/ftx054 (2017). 10.1093/femspd/ftx054 28505248

[2] Sessa R (2017). Effect of bovine lactoferrin on *Chlamydia trachomatis* infection and inflammation. Biochem Cell Biol..

[3] Newman L (2015). Global Estimates of the Prevalence and Incidence of Four Curable Sexually Transmitted Infections in 2012 Based on Systematic Review and Global Reporting. PLoS One..

[4] Bhushan S, Schuppe HC, Fijak M, Meinhardt A (2009). Testicular infection: microorganisms, clinical implications and host-pathogen interaction. J Reprod Immunol..

[5] O’Connell CM, Ferone ME (2016). *Chlamydia trachomatis* Genital Infections. Microb Cell..

[6] Solomon M, Henkel R (2017). Semen culture and the assessment of genitourinary tract infections. Indian J Urol..

[7] Vander Borght, M. & Wyns, C. Fertility and infertility: Definition and epidemiology. *Clin Biochem*. pii: S0009-9120(18)30220-0. 10.1016/j.clinbiochem.2018.03.012 (2018). 10.1016/j.clinbiochem.2018.03.012 29555319

[8] Gimenes F (2014). Male infertility: a public health issue caused by sexually transmitted pathogens. Nat Rev Urol..

[9] Fode M, Fusco F, Lipshultz L, Weidner W (2016). Sexually Transmitted Disease and Male Infertility: A Systematic Review. Eur Urol Focus..

[10] Satta A (2006). Experimental *Chlamydia trachomatis* infection causes apoptosis in human sperm. Hum Reprod..

[11] O’Doherty AM, Di Fenza M, Kölle S, Lipopolysaccharide LPS (2016). disrupts particle transport, cilia function and sperm motility in an *ex vivo* oviduct model. Sci Rep..

[12] Eley A, Hosseinzadeh S, Hakimi H, Geary I, Pacey AA (2005). Apoptosis of ejaculated human sperm is induced by co-incubation with *Chlamydia trachomatis* lipopolysaccharide. Hum Reprod..

[13] Ahmadi, M. H., Mirsalehian, A., Sadighi Gilani, M. A., Bahador, A., Afraz, K. Association of asymptomatic *Chlamydia trachomatis* infection with male infertility and the effect of antibiotic therapy in improvement of semen quality in infected infertile men. *Andrologia*, 10.1111/and.12944 (2018). 10.1111/and.12944 29292525

[14] Moazenchi M (2018). The impact of *Chlamydia trachomatis* infection on sperm parameters and male fertility: A comprehensive study. Int J STD AIDS..

[15] Sellami H (2014). Molecular detection of *Chlamydia trachomatis* and other sexually transmitted bacteria in semen of male partners of infertile couples in Tunisia: the effect on semen parameters and spermatozoa apoptosis markers. PLoS One..

[16] Sobinoff AP (2015). *Chlamydia muridarum* infection-induced destruction of male germ cells and sertoli cells is partially prevented by Chlamydia major outer membrane protein-specific immune CD4 cells. Biol Reprod..

[17] Mruk DD, Cheng CY (2015). The Mammalian Blood-Testis Barrier: Its Biology and Regulation. Endocr Rev..

[18] Iliadou PK, Tsametis C, Kaprara A, Papadimas I, Goulis DG (2015). The Sertoli cell: Novel clinical potentiality. Hormones (Athens)..

[19] Johnson KJ (2014). Testicular histopathology associated with disruption of the Sertoli cell cytoskeleton. Spermatogenesis..

[20] Wen Q (2018). Regulation of Blood-Testis Barrier (BTB) Dynamics, Role of Actin-, and Microtubule-Based Cytoskeletons. Methods Mol Biol..

[21] Taylor-Robinson D, Thomas BJ (1991). Laboratory techniques for the diagnosis of chlamydial infections. Genitourin Med..

[22] Lyons JM, Ito JI, Peña AS, Morré SA (2005). Differences in growth characteristics and elementary body associated cytotoxicity between *Chlamydia trachomatis* oculogenital serovars D and H and *Chlamydia muridarum*. J Clin Pathol..

[23] O’Connell CM (2011). Toll-like receptor 2 activation by *Chlamydia trachomatis* is plasmid dependent, and plasmid-responsive chromosomal loci are coordinately regulated in response to glucose limitation by *C. trachomatis* but not by *C. muridarum*. Infect Immun..

[24] Donati M (2009). *Chlamydia trachomatis* serovar distribution and other concurrent sexually transmitted infections in heterosexual men with urethritis in Italy. Eur J Clin Microbiol Infect Dis..

[25] Twin J (2011). *Chlamydia trachomatis* genotypes among men who have sex with men in Australia. Sex Transm Dis..

[26] Verweij SP (2014). Serovar D and E of serogroup B induce highest serological responses in urogenital *Chlamydia trachomatis* infections. BMC Infect Dis..

[27] Skilton RJ (2018). The *Chlamydia muridarum* plasmid revisited: new insights into growth kinetics. Wellcome Open Res..

[28] Pickett MA, Everson JS, Pead PJ, Clarke IN (2005). The plasmids of *Chlamydia trachomatis* and Chlamydophila pneumoniae (N16): accurate determination of copy number and the paradoxical effect of plasmid-curing agents. Microbiology..

